# P-1954. Clinical, Laboratory, and Biomarker Predictors of 90-Day Mortality in Non-HIV, Non-Transplant Pneumocystis Pneumonia

**DOI:** 10.1093/ofid/ofaf695.2122

**Published:** 2026-01-11

**Authors:** Melat Endashaw, Leland Shapiro, Brent Edwards, Daniel B Chastain, James Maloney, George R Thompson III, Andrés F Henao Martínez

**Affiliations:** Emory Healthcare, Atlanta, GA; Rocky Mountain Regional Veterans Affairs Medical Center, Denver, Colorado; University of Colorado Denver, Department of Medicine, Division of Infectious Diseases, Aurora, Colorado; University of Georgia College of Pharmacy, Albany, GA; Univ. of Colorado Anschutz Medical campus, aurora, Colorado; University of California, Davis Medical Center, Sacramento, California; University of Colorado Anschutz Medical Campus, Aurora, Colorado

## Abstract

**Background:**

*Pneumocystis jirovecii* pneumonia (PJP) poses a growing threat in immunocompromised, non-HIV, non-transplant (NHNT) adults. Clinical features and pathogenesis associated with mortality are poorly characterized. This study explores clinical features and biomarkers reflecting immunologic capacity, inflammation, and pathogen load associated with NHNT PJP 90-day mortality.upda

**Table 1.**
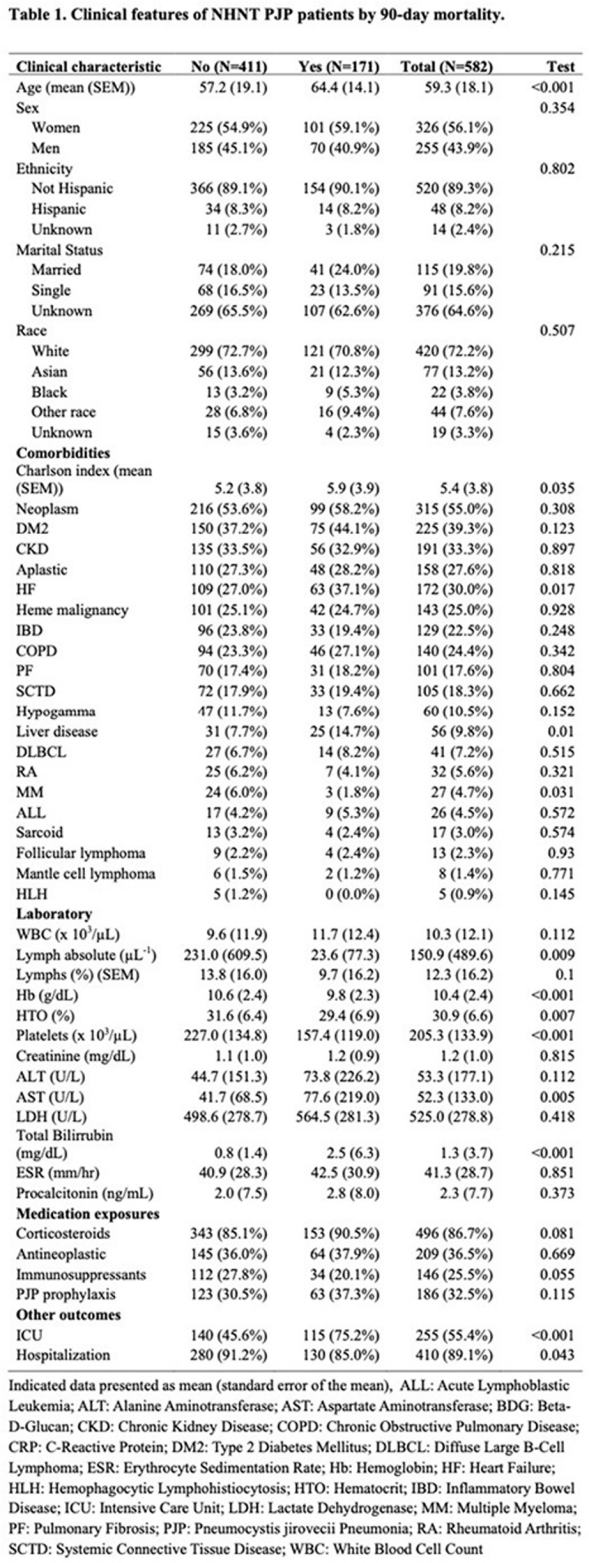
Clinical features of NHNT PJP patients by 90-day mortality

**Figure 1.**
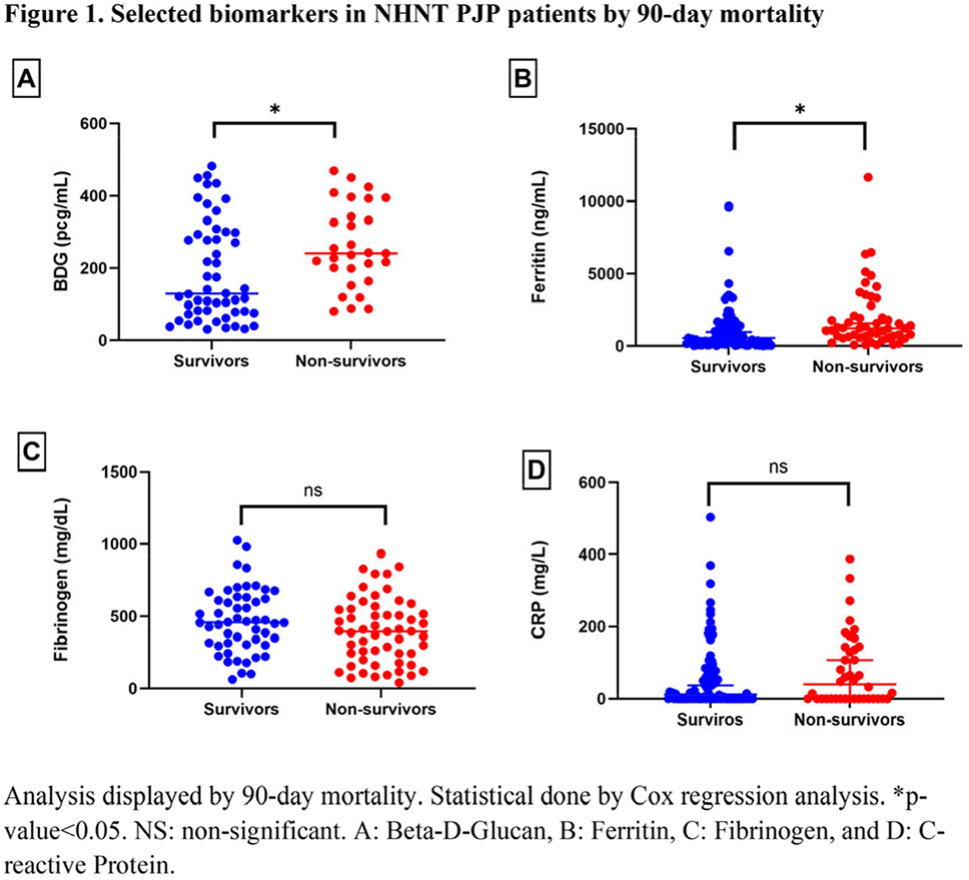
Selected biomarkers in NHNT PJP patients by 90-day mortality

**Methods:**

This retrospective cohort study (2011-2024) used TriNetX, a federated database of anonymized electronic health records. We selected adults with PJP (positive PCR orβ-D-glucan [BDG] results from respiratory samples) who had neither HIV nor history of solid organ transplantation. We compared demographics, comorbidities, immunosuppressant use, and laboratory biomarkers in survivors and non-survivors. Cox regression analysis was used to assess 90-day mortality differences.

**Results:**

In 578 NHNT patients with PJP, 90-day mortality was 29.4%. Non-survivors were significantly older (mean 64.4 ± 14.1 vs 57.2 ± 19.1 years, p< 0.001) and had a higher Charlson comorbidity index (mean 5.9 ± 3.9 vs 5.2 ± 3.8, p=0.035), with increased prevalence of liver disease (mean 14.7% vs 7.7%, p=0.010) and heart failure (mean 37.1% vs 27.0%, p=0.017) (Table 1). Laboratory findings showed lower lymphocyte counts (23.6 ± 77.3 vs 231.0 ± 609.5 µL^-1^, p=0.009), hemoglobin (9.8 vs 10.6 g/dL, p< 0.001), and platelets (157.4 vs 227.0 x 10^3^/µL, p< 0.001) in non-survivors. Elevated total bilirubin was also associated with mortality (2.5 vs 0.8 mg/dL, p< 0.001). Scatter plots by unadjusted Cox regression showed higher serum BDG (300 vs 200 pcg/mL, HR: 20.2 p=0.018) and ferritin (1901.9 vs 1158.5 ng/mL, HR: 1.0, p=0.025) in non-survivors. In contrast, other acute phase reactants fibrinogen and C-reactive protein (CRP) had no significant differences (Figure 1).

**Conclusion:**

In NHNT adults with PJP, 90-day mortality was significantly associated with older age and organ dysfunction. As expected, evidence of immune dysfunction (absolute lymphocyte count) or a biomarker of fungal burden (serum BDG) predicted mortality, and no association with indicators of inflammation (CRP) was detected. Clinicians should prioritize early identification and management of high-risk patients and pursue a model-based approach to NHNT PJP therapeutics.

**Disclosures:**

George R. Thompson III, MD, Astellas: Advisor/Consultant|Astellas: Grant/Research Support|Basilea: Advisor/Consultant|Basilea: Grant/Research Support|Cidara: Advisor/Consultant|Cidara: Grant/Research Support|F2G: Advisor/Consultant|F2G: Grant/Research Support|GSK: Advisor/Consultant|GSK: Grant/Research Support|Melinta: Advisor/Consultant|Melinta: Grant/Research Support|Mundipharma: Advisor/Consultant|Mundipharma: Grant/Research Support|Scynexis: Advisor/Consultant|Scynexis: Grant/Research Support Andrés F. Henao Martínez, MD, MPH, F2: Grant/Research Support|Scynexis: Grant/Research Support

